# Current Knowledge on the Function of α-Methyl Acyl-CoA Racemase in Human Diseases

**DOI:** 10.3389/fmolb.2020.00153

**Published:** 2020-07-14

**Authors:** Gyeyeong Kong, Hyunji Lee, Quangdon Tran, Chaeyeong Kim, Jisoo Park, So Hee Kwon, Seon-Hwan Kim, Jongsun Park

**Affiliations:** ^1^Department of Pharmacology, College of Medicine, Chungnam National University, Daejeon, South Korea; ^2^Department of Medical Science, Metabolic Syndrome and Cell Signaling Laboratory, Institute for Cancer Research, College of Medicine, Chungnam National University, Daejeon, South Korea; ^3^Department of Life Science, Hyehwa Liberal Arts College, LINC Plus Project Group, Daejeon University, Daejeon, South Korea; ^4^College of Pharmacy, Yonsei Institute of Pharmaceutical Sciences, Yonsei University, Incheon, South Korea; ^5^Department of Neurosurgery, Institute for Cancer Research, College of Medicine, Chungnam National University, Daejeon, South Korea

**Keywords:** AMACR, branched-chain fatty acid, cancer development, β-oxidation, lipid metabolism

## Abstract

Branched chain fatty acids perform very important functions in human diet and drug metabolism. they cannot be metabolized in mitochondria and are instead processed and degraded in peroxisomes due to the presence of methyl groups on the carbon chains. Oxidative degradation pathways for lipids include α- and β-oxidation and several pathways. In all metabolic pathways, α-methyl acyl-CoA racemase (AMACR) plays an essential role by regulating the metabolism of lipids and drugs. AMACR regulates β-oxidation of branched chain lipids in peroxisomes and mitochondria and promotes chiral reversal of 2-methyl acids. AMACR defects cause sensory-motor neuronal and liver abnormalities in humans. These phenotypes are inherited and are caused by mutations in AMACR. In addition, AMACR has been found to be overexpressed in prostate cancer. In addition, the protein levels of AMACR have increased significantly in many types of cancer. Therefore, AMACR may be an important marker in tumors. In this review, a comprehensive overview of AMACR studies in human disease will be described.

## Introduction

**FIGURE 1:**
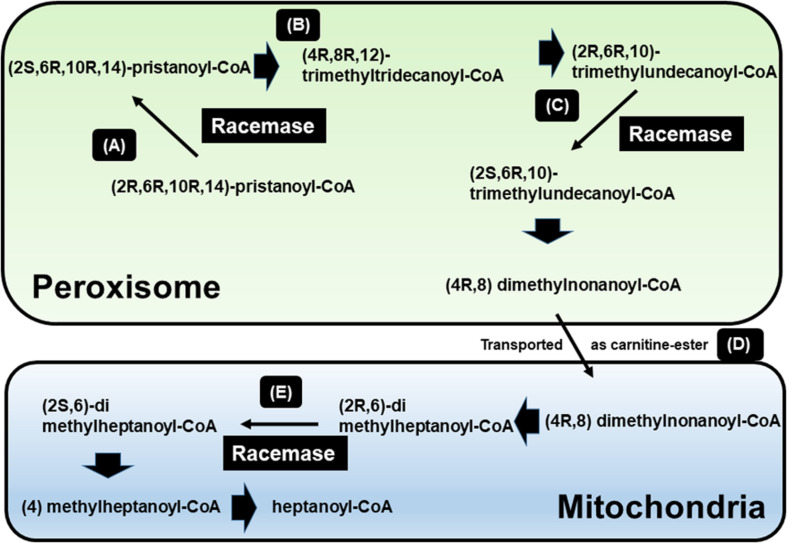
β-oxidation of pristanic acid and racemase activity in peroxisomes and mitochondria. **(A)** (2R,6R,10R,14)-pristanoyl-CoA exist the half of natural pristanoyl-CoA. The limited action of branched-chain acyl-CoA oxidase (first enzyme in β-oxidation), (R)-stereoisomer should be changed to its (S)-stereoisomer by racemase. **(B)** The intermediate (4R,8R,12) trimethyltridecanoyl-CoA can be further β-oxidized to (2R,6R,10)-trimethylundecanoyl-CoA. **(C)** Again, the resulting (R)-acyl-CoA then also need to be changed to (S)-stereoisomer by racemase. **(D)** After another β-oxidation occurs, (4R,8)-dimethylnonanoyl-CoA is moved to mitochondria from peroxisome via acyl-carnitine ester formation. **(E)** After another β-oxidation occurs, the intermediate (2R,6)-dimethylheptanoyl-CoA also need to be (S)-stereoisomer by racemase [adapted from Ferdinandusse et al. (2000b)].

The role of branched chain fatty acids is very important for drug metabolism such as ibuprofen and human diet. They occur mainly from the catabolism of isoprenoids, such as phytanic acid (3,7,11,15-tetramethyl hexadecanoic acid), a derivative of the chlorophyll component phytol (Schmitz et al., 1995). Most of the branched chain fatty acids cannot be metabolized immediately in the mitochondria due to the methyl group of the carbon chain, but in peroxisomes (Darley et al., 2009). In eukaryotes, like animals and plants, peroxisomes are universal organelles. Peroxisome target signals allow for the delivery of proteins to intact peroxisomes. All peroxisomes contain more than 50 enzymes, including oxidases and oxygenases, and the content of peroxisomes varies by organism and cell type (Mukherji et al., 2003). A member of the CAIB-BAIF CoA transferase family, α-methyl acyl-CoA racemase (AMACR) (White et al., 1988) catalyzes steric conversion of α-methyl protons, which proceeds to β-oxidation of branched chain fatty acids in mitochondria and peroxisomes (Daugherty et al., 2007; Autio et al., 2014; Shapovalova et al., 2018). AMACR catalyzes the reversal of 2R to 2S of fatty acyl CoA esters at the 2 position and controls the entry of metabolites to peroxisomes. The sequence of the enzyme confines it to two organelles (N-terminal mitochondrial target signal and C-terminal peroxisomal targeting signal-1) (Amery et al., 2000; Ferdinandusse et al., 2000b; Kotti et al., 2000; Darley et al., 2009). A decrease in AMACR activity leads to an increase in R-2 methyl fatty acids, which leads to human nervous system disorders (Ferdinandusse et al., 2000a). AMACR depletion in mice increased bile acid precursor levels and decreased bile acid levels (Savolainen et al., 2004). In addition, changes in the mRNA levels of some lipid metabolizing enzymes were also observed, but no pathological symptoms were observed in the absence of branched-chain lipids. Interestingly, supplementing the diet with phytol, a lipid precursor of phytanic and pristanic acids, resulted in a disease state in AMACR knockout (KO) mice, whereas no such changes were observed in control mice (Savolainen et al., 2004). Therefore, the deficiency of AMACR or protein inactivation causes neurological disorders (Ferdinandusse et al., 2000a; McLean et al., 2002; Thompson et al., 2008) due to the accumulation of branched-chain fatty acids and is also associated with various peroxisome disorders (Assoum et al., 2010; Dick et al., 2011). AMACR catalyzes the racemization of acyl-CoA’s of α-methyl branched acids such as di-trihydroxycoprostanoic acids (D/THCA) (Ferdinandusse et al., 2000b; Wanders et al., 2001). Due to the stereospecific properties of acyl-CoA oxidase that act on α-methyl acyl-CoA in human hepatic peroxisomes, AMACR is essential for chain shortening when β-oxidation of the substrate occurs. In patients with AMACR deficiency, increased levels of pristanic acid have been observed (Haugarvoll et al., 2013). AMACR is an essential Enzyme analysis has shown that AMACR catalyzes chemical reactions and is essential for the β-oxidation of branched-chain fatty acid and bile acid intermediates (Clarke et al., 2004; Figure 1; Autio et al., 2014). Natural substrates of AMACR include (2R)/(2S)-pristanoyl coenzyme A and the bile acid precursor molecule (25R)/(25S)-trihydroxy cholestanoyl coenzyme A. The enzyme catalyzes both directions. The steric conversion of α-methyl protons (S-> R, R-S) via 1.1-proton transfer is thought to proceed through the enolate intermediate (Schmitz et al., 1995; Wilson et al., 2011). AMACR has also been reported to be important in many human diseases. Patients with Zellweger syndrome (Ferdinandusse et al., 2001) have no AMACR activity and patients with AMACR mutations (S52P and L107P) accumulate toxic levels of branched-chain fatty acids in the blood, causing neuropathy similar to Refsum disease (Ferdinandusse et al., 2000a; Wilson et al., 2011; Herzog et al., 2017). Jiang et al. found high expression of AMACR protein and mRNA in prostate cancer (Jiang et al., 2001), demonstrating the usefulness of AMACR as a diagnostic marker for prostate cancer (Walsh, 2002; Halsey et al., 2010).

## The Role of AMACR in Mitochondria

**FIGURE 2:**
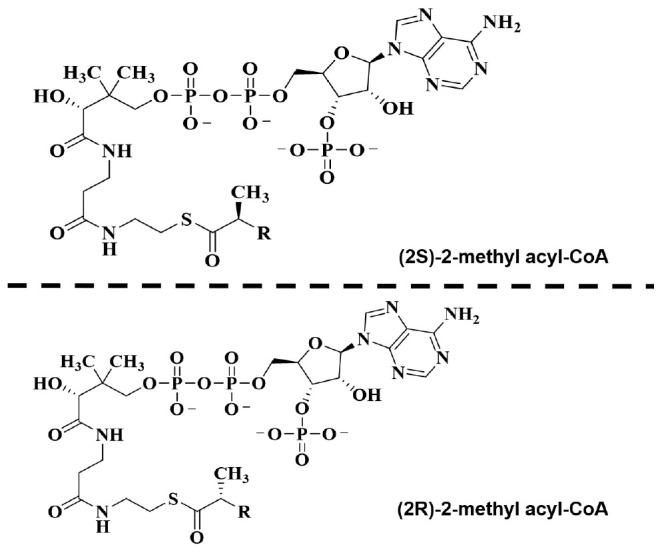
Illustration for the oxidation step of (3R)- and (3S)-phytanic acid as resulting from normal diet and (25R)-trihydroxy cofluorocarbonate (THCA) biosynthesized from liver-derived cholesterol.

α-methyl acyl-CoA racemase catalyzes racemization of α-methyl branched carboxylic acid coenzyme A thioester (Kotti et al., 2000) and converts the (S)-isomer of (2R)-methyl branched-chain fatty acyl-CoA. It also induces these stereoisomers to proceed through the β-oxidation pathway. AMACR converts (2R)-methyl branched chain fatty acyl-CoA into (S)-stereoisomers to catalyze these stereoisomers to proceed through the β-oxidation pathway. The expression of AMACR is important for the oxidative metabolism and biosynthesis of these branched chain fatty acids and bile acids, and the expression of AMACR is observed in peroxisomes and mitochondria (Schmitz et al., 1995; Pedersen et al., 1996; Van Veldhoven et al., 1996, 1997; Amery et al., 2000). AMACR induces β-oxidation of branched chain fatty acids and fatty acid derivatives by catalyzing the conversion of several (2R) methyl-branched chain fatty acid acyl-CoA molecules to the (S) stereoisomer (Ferdinandusse et al., 2000b). Side-chain cleavage of bile acids and β-oxidation of methyl-branched fatty acids occur in peroxisomes. However, the ratio of AMACR activity occurring in peroxisomes and mitochondria was found to vary between species. In human tissues, 80–90% of AMACR activity have been shown to be involved in a uniformly distributed peroxisomes (Schmitz et al., 1995). In mice and Chinese hamsters, AMACR is distributed almost equally with peroxisomes and mitochondria (Schmitz et al., 1994). However, AMACR activity in rats was found only in mitochondria. The molecular mechanism underlying the distribution of AMACR is not yet known, only one cDNA sequence for the enzyme has been found in mice and humans (Kotti et al., 2000). Branched-chain fatty acids may be linked to cancer because the production of reactive oxygen species causes oxidative stress and DNA damage. This hypothesis is supported by a study that showed that ibuprofen (a non-oxidative substrate of AMACR) protects against cataracts (Lloyd et al., 2008). In hepatocytes, AMACR catalyzes the conversion of R- to S- from pristanoyl-CoA and C27-villeayl-CoA. This is the only stereoisomers that can undergo β-oxidation. Derivatives of these branched-chain fatty acids are transported to mitochondria and further degraded to produce energy. Since most malignant tumors produce energy using fatty acids for growth, increased β-oxidation of branched-chain fatty acids has been suggested to generate transformed cells with unique metabolic benefits (Zhang et al., 2009). Mitochondrial hydroxylation in the condensed C-26 of cholesterol provides the (25R)-diastereomer of di- and trihydroxy coprostanoic acid (THCA). Therefore, enzymes play an important physiological role in the biosynthesis of bile acids. In addition, the reaction catalyzed by peroxide iso-oxidase, which initiates β-oxidative degradation of the side chains, is specific for the (25S)-stereoisomer. Thus, to connect the two pathways, it is necessary to reverse the composition of C-25 and efficiently racemase THCA-CoA with AMACR (Figure 2; Kotti et al., 2000).

## The Role of AMACR in Disease

α-methyl acyl-CoA racemase is associated with many of the diseases mentioned earlier. Patients with Zellweger syndrome (Ferdinandusse et al., 2001) have low AMACR activity and patients with AMACR mutations (S52P and L107P) accumulate toxic levels of branched-chain fatty acids in the blood, causing neuropathy similar to Refsum disease (Ferdinandusse et al., 2000a; Wilson et al., 2011; Herzog et al., 2017). For example, AMACR deficiency is a disorder that begins in adults and slowly worsens, causing a variety of neurological problems. People with AMACR deficiency develop cognitive decline, seizures, and migraine headaches. Stroke-like brain dysfunction (encephalopathy) can occur acutely, resulting in loss of consciousness and damage to the brain. Other features of AMACR deficiency include weakness and loss of limb sensations due to nerve damage (sensorimotor neuropathy) and muscle stiffness (spasticity), and difficulty with coordination of movement (ataxia) (Ferdinandusse et al., 2000a; Savolainen et al., 2004; Kanwal et al., 2018). In addition, fever may develop in the retina behind the eye, which can cause vision problems. AMACR-defective mice exhibited the same unconjugated and conjugated bile acid precursors (C27) and reduced bile acids (C24) as patients with AMACR deficiency. Despite the disturbed bile acid pattern, the phenotype of AMACR-KO mice was clinical, and no symptoms were visible. However, AMACR-KO mice exhibited intolerance to phytol feed, indicating that AMACR is essential for the removal of methyl-branched fatty acids and derivatives thereof (Savolainen et al., 2004). Also, in the end stage of renal disease, high expression of AMACR was observed in papillary kidney adenoma and multifocal papillary carcinoma (Kansal et al., 2018). Mutations in the AMACR gene caused various disorders identified by homozygous S52P mutations. Mutations in the AMACR gene are associated with sensorimotor neuropathy, muscle stiffness, and difficulty with coordination of movement (Lloyd et al., 2008).

## AMACR Function in Cancer

**FIGURE 3:**
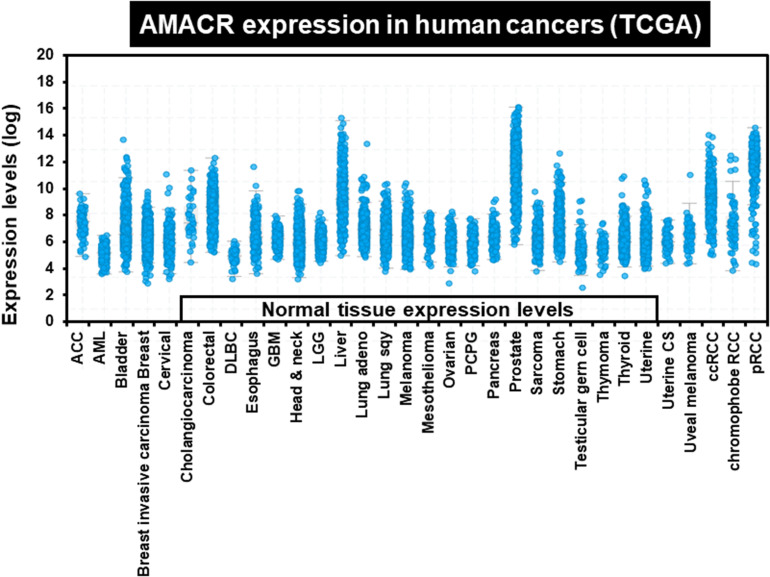
Increased expression of AMACR in 32 different human cancers. AMACR expression levels in various human cancers identified by TCGA statistics. High expression of AMACR was observed in 32 different cancers [adapted from Lee (2019)].

**FIGURE 4:**
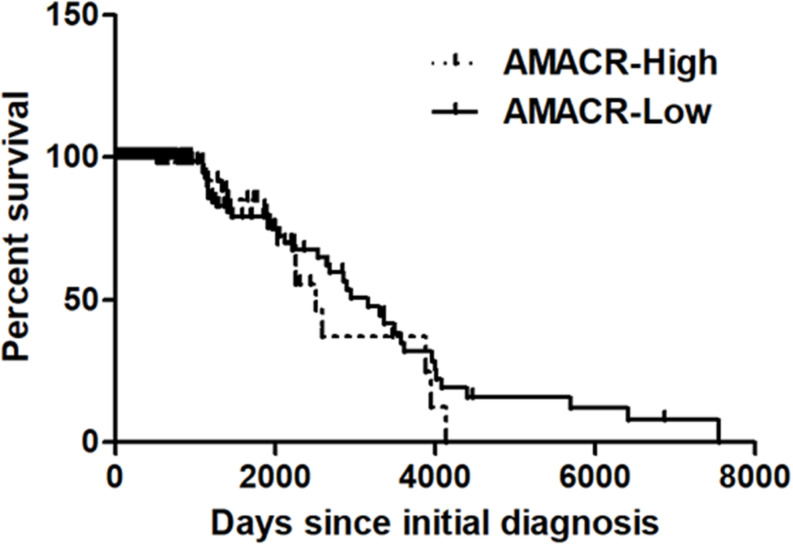
Kaplan–Meier’s analysis for overall survival based on AMACR expression in glioma specimens of the REMBRANDT cohort. The Kaplan–Meier curve compares the level of AMACR mRNA expression with overall survival. *P* values were obtained using the log-rank test, and hazard ratios (HRs) with 95% confidence intervals (CIs) were determined using the aid of a univariate Cox’s regression model. GBM dataset; http://www.betastasis.com/glioma/tcga_gbm/) [adapted from Lee (2019)].

**FIGURE 5:**
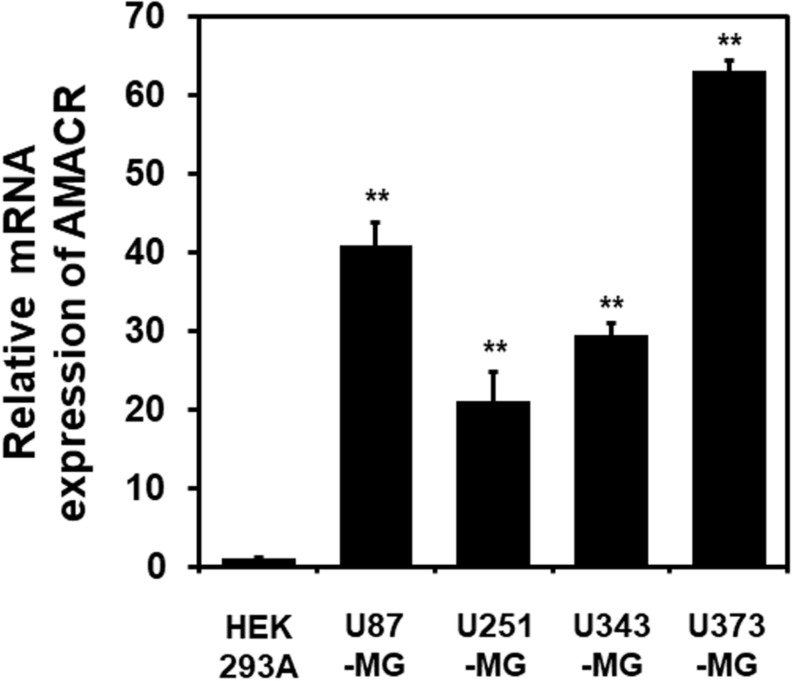
Elevation of AMACR mRNA levels in human glioblastoma cell lines. Total RNA from each glioblastoma cell line was extracted and analyzed using human AMACR specific primers by real-time quantitative polymerase chain reaction (qPCR). The results are presented as the means ± SD obtained from three independent experiments. Expression of AMACR mRNA was greatly increased in all glioblastoma cell lines. *, *p* < 0.05 [adapted from Lee (2019)].

One of the most notable diseases in modern society is cancer. Cancer destroys normal tissues, stops working and causes death through metastasis to other cells. These cancer cells multiply using various signaling pathways (Sanchez-Vega et al., 2018; Jung, 2019). Cancer cells have many causes, but are usually caused by smoking, excessive meat intake, drinking and infection. Red meat and dairy products are rich in branched-chain fatty acids (Lin et al., 2007). β-oxidation of these branched-chain fatty acids requires the enzyme AMACR located in mitochondria and peroxisomes. The AMACR promoter can induce cancer-specific expression of a reporter gene (Shukla et al., 2017). AMACR expression is high in several types of cancers (Thul et al., 2017), including gastric cancer (Nozawa et al., 2012), ovarian cancer (Noske et al., 2011), renal cell carcinoma (Eichelberg et al., 2013), and hepatocellular carcinoma (Figure 3; Yu et al., 2019). Increased expression of AMACR also affects the survival of cancer patients (Figure 4; Lee, 2019). We also confirmed the expression of AMACR in human glioblastoma cell lines (U87-MG, U251-MG, U343-MG, and U373-MG) (Figure 5). mRNA levels of AMACR in glioblastoma showed >60-fold higher expression than the control (Lee, 2019). AMACR may provide metabolic support for tumor growth through the multi-step β-oxidation of certain branched-chain fatty acids. It is also possible that peroxisomal AMACR activity is increased to activate β-oxidation and increase cellular peroxide production. Degradation of the (2)-methyl acyl-CoA ester by peroxisomal β-oxidation requires conversion to the (S)-stereoisomer. AMACR serves to convert (2R)-methyl acyl-CoA esters into (2S)-methyl acyl-CoA epimers (Van Veldhoven et al., 1996; Yevglevskis et al., 2017). This increases the production of cell peroxides, regardless of catalase expression. As a result, it promotes DNA damage in prostate cancer cells, leading to an oxidative environment (He et al., 2018). Indeed, lowering the expression of AMACR in cancer cells via si-RNA has been shown to reduce the growth of cancer cells. One of the inhibitors found to date, N-Dodecyl-N-methylcarbamoyl-CoA 1 is a representative inhibitor (Yevglevskis et al., 2017). In other prostate cells expressing AMACR (LAPC4, LNCaP, and PC3), ebselen and ebselen are selective covalent inhibitors, and 2-trifluoromethyl tetradecanoyl-CoA and E-13-iodo-2 methylenetridec-12-enoyl-CoA as competitive inhibitors Was reported (Carnell et al., 2007, 2013; Morgenroth et al., 2011; Festuccia et al., 2014; Yevglevskis et al., 2018, 2019; Petrova et al., 2019). These results suggest that ibuprofen and other nonsteroidal anti-inflammatory drugs are effective in preventing prostate and colon cancer, and this effect may be caused by AMACR inhibition based on previous studies (He et al., 2018).

## Future Studies of AMACR

**FIGURE 6:**
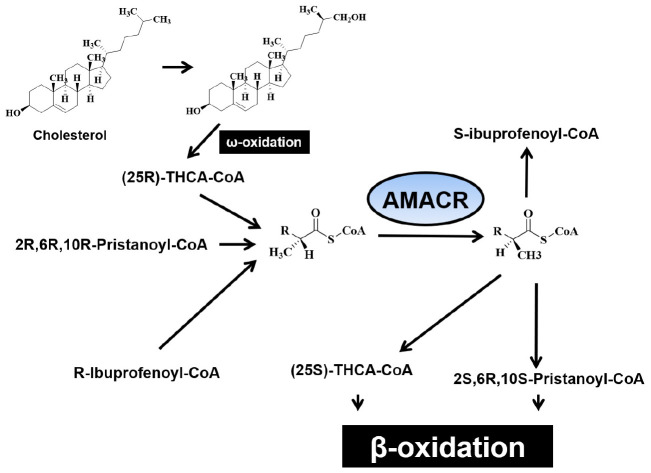
Structure of α-methyl-branched-chain fatty acyl-CoA esters.

**FIGURE 7:**
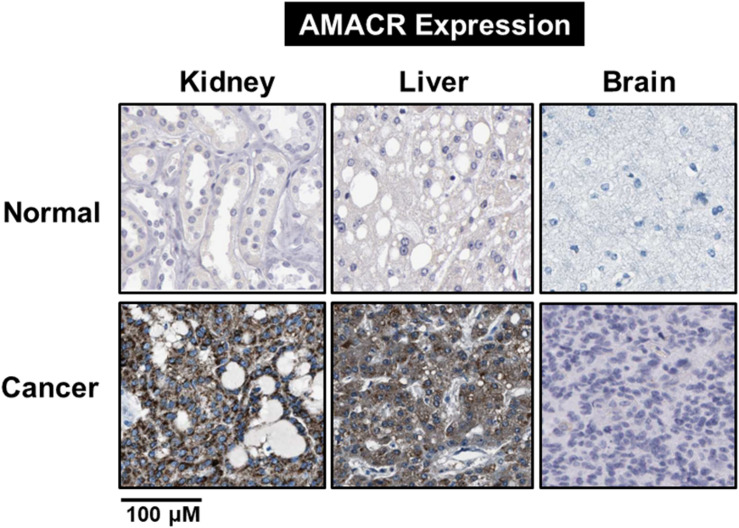
Identification of AMACR expression in normal and cancer cells. Expression of AMACR detected by immunohistochemistry in normal and cancer cells. High expression levels of AMACR were confirmed in kidney, liver, and brain cancer. Scale bars represent 100 μM. The representative images are from Protein-atlas projects [adapted from Uhlen et al. (2017)].

**FIGURE 8:**
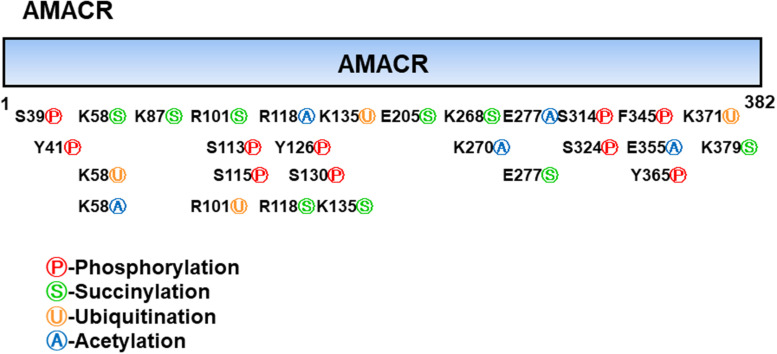
Post-translational modifications of α-methyl acyl-CoA racemase (AMACR). AMACR domains range from 1 to 382 and many post-translational modification sites exist, including phosphorylation (red: S39, Y41, S113, S115, Y126, S130, S314, S324, F345, and Y365), succinylation (green: K58, K87, R101, R118, K135, E205, K268, E277, and K379), ubiquitination (yellow: K58, R101, K135, and K371), and acetylation (blue: K58, R118, K270, E277, and E355) [adapted from Hornbeck et al. (2015)].

Branched-chain fatty acids are critical mediators of human nutrition and drug metabolism. They occur mainly from the catabolism of isoprenoids, and the presence of phytanic acid (3,7,11,15-tetramethyl hexadecanoic acid), derived from chlorophyll-based phytol, is also an important risk factor for prostate cancer. Metabolic pathways responsible for fatty acid degradation are upregulated in cancer. AMACR is expressed in mitochondria and peroxisomes and acts as a regulator of β-oxidation. AMACR is involved in β-oxidation of branched-chain fatty acids through the catalytic conversion of (2R)-2-methyl acyl-CoA esters to (2S) (Figure 6; Morgat et al., 2019). Recent studies have shown that numerous cancer cells have higher expression of AMACR compared with normal tissue (Figure 7; Uhlen et al., 2017). However, the pathological relationship between β-oxidation and branched-chain fatty acids in cancer cells with high expression of AMACR is unclear. One hypothesis is that the generation of reactive oxygen species induces oxidative stress, leading to DNA damage. This theory is supported by experimental results showing that ibuprofen (a non-β-oxidizable substrate of AMACR) protects against cataracts. Preliminary epidemiological studies reported to date indicate that phytanic acid can be used to treat Refsum disease. In addition, the function of AMACR in prostate cancer is very important. Indeed, the growth and maintenance of prostate tissue depends on the androgens produced by the testes and adrenal glands, and intracellular androgen receptor (AR) signaling plays an important role in the formation and development of prostate cancers. AMACR mRNA detected in cancer tissues increased by 682 times compared to normal tissue in patients with prostate cancer, and increased by 11.5 times for AR mRNA (Andersson et al., 1991; Alinezhad et al., 2016). According to the reported results, the expression of AR and IGF-1 in prostate cancer decreased as the expression of AMACR decreased (Takahara et al., 2009). In addition, as a result of sequencing the promoter portion of AMACR in prostate cancer, 17 sequence variants were identified. Based on these results, it is estimated that AMACR expression and genetic variation may adversely affect prostate cancer (Zheng et al., 2002; Thornburg et al., 2006). Recent studies have shown that phytanic acid intake is effective in men at risk for developing prostate cancer (Lloyd et al., 2008). Based on these results, we hypothesize that this diet can be used for the prevention of breast cancer, ovarian cancer, and other major cancers. According to the results reported so far, target drugs that inhibit the expression of AMACR in prostate cancer are typically ibuprofenoyl-CoA derivatives (Petrova et al., 2019), and 2-(phenylthio)propanoyl-CoA derivatives (Yevglevskis et al., 2018). All of these have been reported to be involved in the growth and survival of cancer cells by inhibiting the expression of AMACR in cancer cells. However, most studies have been conducted in prostate cancer. In order to confirm the genetic mutation of AMACR in many types of cancer and its expression in cancer cells, it is necessary to study the molecular mechanism of AMACR. Basically, AMACR has many post-transcriptional sites, (Figure 8; Hornbeck et al., 2015) but not many studies have not been conducted on their role. It is thought that the post-transcriptional modification site of AMACR may be important for the function of AMACR expressed in many cancers. In addition, identification of multiple splice variants of AMACR is necessary to confirm the correct association between branched chain fatty acids and cancer. In addition, the identification of these multiple splice variants will help predict the pathological and physiological roles of AMACR. Further studies are required to determine if AMACR splice variants have a distinct catalytic activity and play a role in normal and cancer cells. It will also be important to identify the changes in cancer cells caused by these splice variants and broaden diagnostic methods using specific splice modifications or antibodies.

## Author Contributions

GK, HL, JiP, S-HK, and JoP contributed to the conception and design of the study. GK, QT, JiP, and CK organized the database. GK wrote the first draft of the manuscript. GK, HL, QT, CK, SK, and JiP wrote sections of the manuscript. All authors contributed to manuscript revision, read and approved the submitted version.

## Conflict of Interest

The authors declare that the research was conducted in the absence of any commercial or financial relationships that could be construed as a potential conflict of interest.
