# Corrigendum: Current Knowledge on the Function of α-Methyl Acyl-CoA Racemase in Human Diseases

**DOI:** 10.3389/fmolb.2021.639164

**Published:** 2021-03-25

**Authors:** Gyeyeong Kong, Hyunji Lee, Quangdon Tran, Chaeyeong Kim, Jisoo Park, So Hee Kwon, Seon-Hwan Kim, Jongsun Park

**Affiliations:** ^1^ Department of Pharmacology, College of Medicine, Chungnam National University, Daejeon, South Korea; ^2^ Department of Medical Science, Metabolic Syndrome and Cell Signaling Laboratory, Institute for Cancer Research, College of Medicine, Chungnam National University, Daejeon, South Korea; ^3^ Department of Life Science, Hyehwa Liberal Arts College, LINC Plus Project Group, Daejeon University, Daejeon, South Korea; ^4^ College of Pharmacy, Yonsei Institute of Pharmaceutical Sciences, Yonsei University, Incheon, South Korea; ^5^ Department of Neurosurgery, Institute for Cancer Research, College of Medicine, Chungnam National University, Daejeon, South Korea

**Keywords:** AMACR, branched-chain fatty acid, cancer development, B-oxidation, lipid metabolism

In the original article, there was a mistake in Figure 2 as published. The cholesterol structure should be the chiral for omega oxidation, instead of the chiral structure used in the figure; as both groups are methyl, we mentioned the oxidation of omega by adding CH2OH to carbon 15 to create a chiral center. The corrected Figure 2 appears below.

**FIGURE 2:**
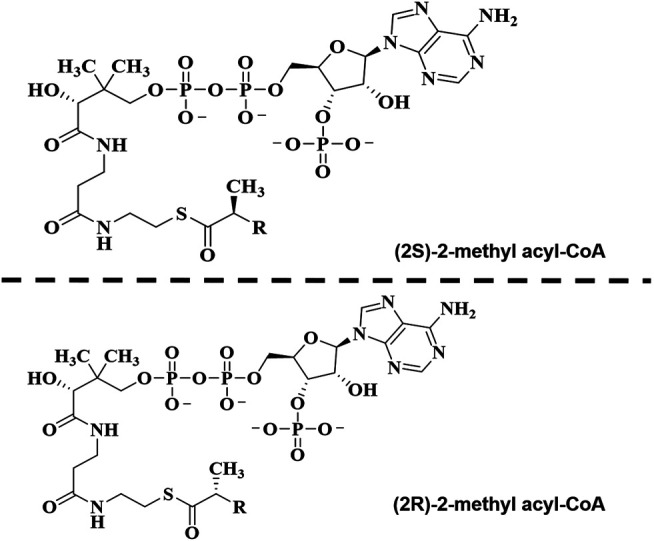
Illustration for the oxidation step of (3R)- and (3S)-phytanic acid as resulting from normal diet and (25R)-trihydroxy cofluorocarbonate (THCA) biosynthesized from liver-derived cholesterol.

Furthermore, in the original article, there was a mistake in the legend for Figure 6 as published. In the thioester part of the AMACR structure, the keto group was mistakenly omitted. The correct Figure 6 and legend appear below.

**FIGURE 6:**
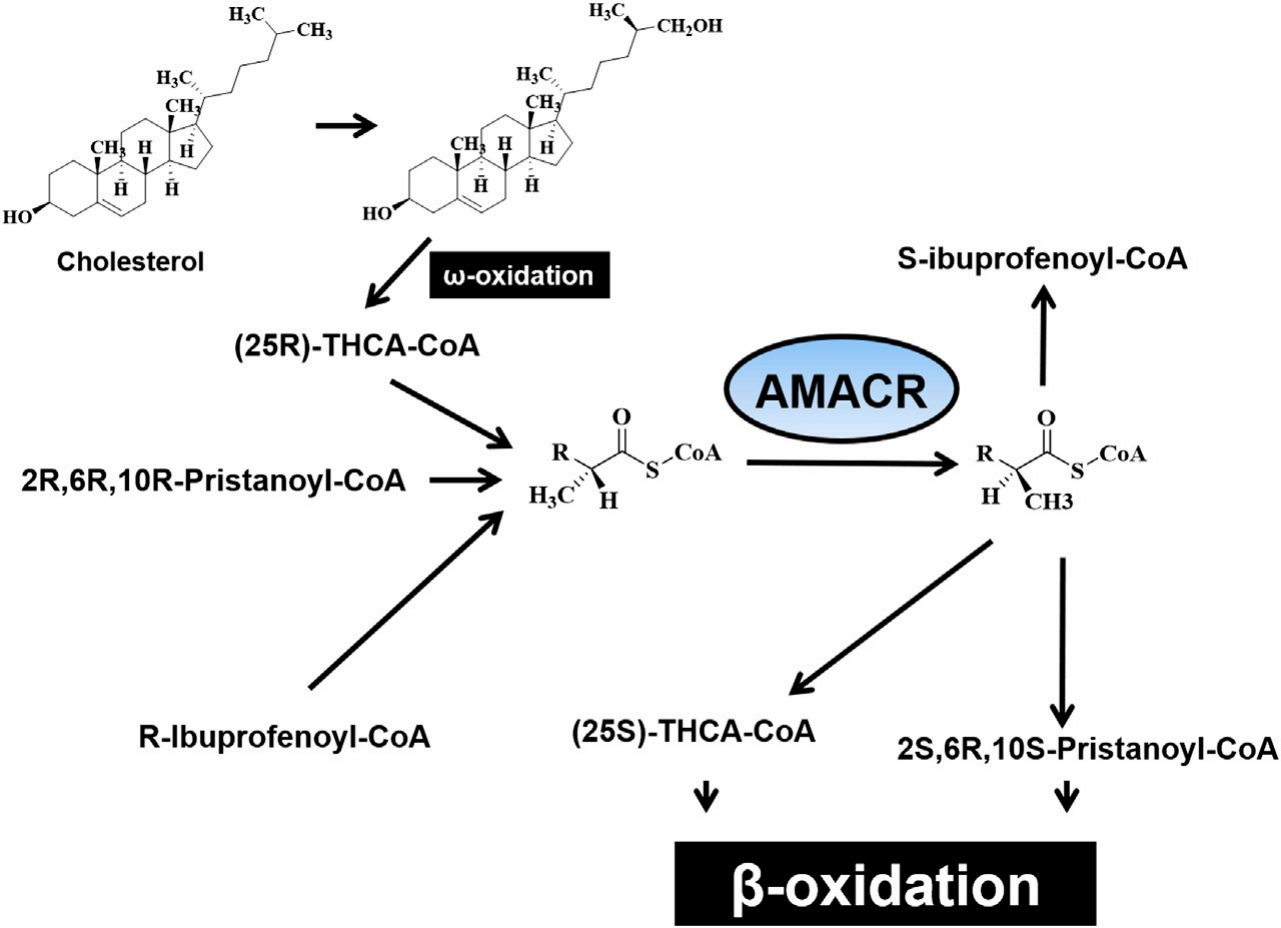
Structure of α-methyl-branched-chain fatty acyl-CoA esters.

The authors apologize for this error and state that this does not change the scientific conclusions of the article in any way. The original article has been updated.

